# Identification of a Novel Haplotype Associated with Roan Coat Color in American Quarter Horses

**DOI:** 10.3390/ani15162386

**Published:** 2025-08-14

**Authors:** Robin E. Everts, Rachael Caron, Gabriel Foster, Kaitlyn McLoone, Laura Simiele, Katie Martin, Samantha A. Brooks, Christa Lafayette

**Affiliations:** 1 Etalon Inc., Menlo Park, CA 94025, USA; rcaron@etalondx.com (R.C.); gfoster@etalondx.com (G.F.); kmcloone@etalondx.com (K.M.); lsimiele@etalondx.com (L.S.); khoefs@etalondx.com (K.M.); 2 Department of Animal Sciences, UF Genetics Institute, University of Florida, Gainesville, FL 32611, USA; samantha.brooks@ufl.edu

**Keywords:** horses, roan, KIT, American Quarter Horse

## Abstract

Roan coat color is described as the dispersion of white hairs within an otherwise solid background-color coat. This phenotype is primarily expressed on the body of the horse, with the head and legs exhibiting few or no white hairs. Previous studies mapped the locus for roan to the *KIT* region and observed linked variants in a small number of breeds. Recently, we reported evidence for two independent haplotypes, *RN1* and *RN2*, in the *KIT* region, which account for approximately 38% and 36% of roan horses, respectively. In the current report, using whole genome sequencing for unknown roan samples. We present a third novel haplotype, *RN3*, found in American Quarter Horses, that accounts for an additional 30% of American Quarter Horses negative for *RN1* and *RN2* that display the roan phenotype. Within this haplotype, we observe a variant chr3:79656505 (G > A), which we believe is a founder allele for the RN3 haplotype. In our sample set of horses, these three haplotypes account for more than 95% of the American Quarter Horse population studied and about 50–60% of roan horses in other breeds. Using whole genome sequencing of distantly related animals with a particular phenotype, together with a larger number of control horses, improves the odds of finding linked and/or causative variants.

## Simple Summary

A roan pattern is described as a horse with a solid-colored coat interspersed with white hairs of varying intensities throughout the body, barrel, hips, chest, and upper legs. Often few or no white hairs are present on the head and legs. Previous studies have mapped the roan locus to the *KIT* region in a small number of breeds. In a recent report, we presented evidence for two independent haplotypes, *RN1* and *RN2*, in the *KIT* region, which account for approximately 74% of tested roan horses. In the current report, using whole genome sequencing of two roan samples not containing an *RN1* or *RN2* haplotype, we present a third haplotype, *RN3*, found in American Quarter Horses. This haplotype accounts for approximately 75% of American Quarter Horses which display the roan phenotype that were negative for *RN1* and *RN2*. In our sample set of horses, these three haplotypes account for 80–90% of roan horses and 90–95% of the Quarter Horse population analyzed in this study.

## 1. Introduction

Roan is considered a variant in the *KIT* gene region, and it is inherited as a dominant trait. It acts epistatically with *ASIP* and *MC1R*, causing white hairs to be expressed throughout the base-coat color of the animal’s body [1]. Roan coat color in other species, such as cattle, pig, sheep, goat, and alpaca, is also associated with the *KIT* or *KITLG* genes [2]. Variants within the *KIT* gene region have been found to be associated with roan for Noriker horses [3] and Icelandic horses [4]. Recently, we published a study analyzing multiple breeds, wherein we validated the original linked variant *RN1*, found by Grilz-Segers (2020) in Noriker horses, while discovering a second linked variant, *RN2* [5], as well. That study showed that the *RN1* haplotype is present in multiple breeds, and the *RN2* haplotype is present mostly in breeds of American heritage. In addition to roan, other *KIT* polymorphisms cause different white spotting phenotypes in horses, such as the Tobiano, Sabino, and Dominant White variants [6]. In our previous paper, we suggested using whole genome sequencing to discover other haplotypes for roan [5]. This manuscript presents the first new roan haplotype we have discovered in this way, and, in doing so, demonstrates the usefulness of this methodology. As many breeders use coat-color genotypes to inform breeding decisions, this new roan haplotype may aid breeders in selecting horses that will provide phenotypes closer to expectation.

## 2. Materials and Methods

We used genomic DNA for a set of horses (*n* = 16), consisting of five *RN1/RN1*, two *RN2/RN2*, two *RN2/n*, one *RN1/RN2,* two roan horses without a known genotype, and five non-roan horses. The two unknown roan horses were picked based on them having the same roan stallion three generations prior, which could improve the chances of finding a common cause. All sixteen samples were whole-genome sequenced using a commercial service to 40× average depth per genome (WGS) (Seqmatic, Fremont, CA, USA). Subsequently, the raw sequence data was aligned to the horse reference genome EquCab 3.0 [7] using BWA-MEM2 [8] with default parameters. IGV version 2.16.2 [9] was used to screen the *KIT* region on chromosome 3 in the resulting bam files for the presence of new variants that can potentially distinguish *RN3* from *RN1*, *RN2*, and non-roan samples in the case–control dataset, under the assumption that *Roan* is a dominant trait.

The selected variants were tested for associations using Fisher’s exact test in a set of 82 roan horses and 223 non-roan horses, including a small number of parental animals that were genotyped with our standard workflow [5]. In short, 20–30 intact hairs (incl follicles) were pulled from the mane or tail, and genomic DNA was extracted from these hair follicle samples using the Puregene Extraction Kit according to the manufacturer’s protocol (QIAGEN, Inc., Germantown, MD, USA) or were retrieved from previously stored and extracted genomic DNA, where applicable. All DNA samples were then sequenced using hybrid capture using paired-end 2 × 150 bp sequencing on a NextSeq1000 instrument (Illumina, San Diego, CA, USA). Biotinylated probes were used to pull out sequences overlapping variants of interest. Sequences with base call quality scores ≥ Q30 and sequencing depths > 40× for all of the assayed regions were aligned to the horse reference genome (EquCab3.0). Selected DNA variants in the region of interest passing the statistical threshold were then tested for putative co-segregation and were assigned into “roan haplotypes” using Fisher’s exact test and Plink (1.90b7.1) [10], with the—r2 option being used to test for linkage between markers and coat color.

For roan horses, coat color was confirmed through owner-provided pictures, owner outreach for coat color confirmation, or online pedigree search tools, e.g., https://www.Allbreedpedigree.com, accessed on, 15 June 2025 and https://quarterhorseresource.com/, accessed on 15 June 2025. For the roan horses selected for sequencing, pedigrees were obtained, and the presence of the roan phenotype was verified in at least one parent in each generation, tracing back to the earliest ancestor with recorded coat color. Additionally, every roan horse within this pedigree was required to have at least one roan parent. For non-roan horses, only bay, black, and chestnut horses with pictures that verified their phenotype were selected from our database. Horses with dilution haplotypes *Cream* or *Pearl* (*SLC45A2*) or *Champagne* (*SLC36A1*) were excluded, as the dilution phenotype could potentially obscure the roan phenotype. No outreach was performed unless the color predicted by the genotypes did not match written records. Criteria for horses with the roan coat color were the same as described in [2], with horses displaying “a mixture of white and colored hair” over the body, excluding the head and lower legs.

## 3. Results and Discussion

In our previous study [5], we observed that the linked markers for other Roan haplotypes could lie distant from *KIT*—in this case, 38–110 kb, outside of the coding region. For this reason, we used whole genome sequencing to discover the novel variants associated with unexplained Roan haplotypes. We included, in the analysis, several *RN1/RN1*, *RN2/RN2, RN1/RN2, RN2/n*, and unknown roan horses, as well as five non-roan horses (Table 1), to identify variants that segregated between roan and non-roan horses. We included the *RN1* and *RN2* samples to ascertain if the same SNPs as described previously were called, thus confirming our procedures and their association with roan. The two unknown roan horses were selected for sequencing, as both inherited their roan phenotype, based on pedigree analysis, from the same horse, the Kitch Roan mare, through two different sons of Zippos Mr Good Bar. Using two horses related through a single roan great-grandparent, it was estimated that only 1–3% of the DNA would be identical by descent, decreasing the likelihood of finding common variants between them due to chance rather than due to the presence of an *RN* haplotype in both horses. This strategy therefore improved our odds of finding variants associated with roan. As we assumed the unknown roan horses possessed a different haplotype, and thus different variants, we assumed that we could use all *RN1*, *RN2*, and non-roan horses as control samples. As the number of control horses was limited in this proof-of-principle study, we assumed that our results could also pick up false positive variants.

Using IGV, we screened the *KIT* region and surrounding area for variants specific to our two unknown roan samples and that were absent in the *RN1*, *RN2*, and non-roan samples. From these variants, we selected 12 variants, seemingly in linkage disequilibrium with roan, that segregated separately from *RN1* and *RN2* and were not present in the five WGS non-roan horses. We then designed hybrid capture probes for five of these variants, spaced throughout the region of interest, and analyzed these on 82 roan horses without the *RN1* or *RN2* haplotypes and on 223 non-roan horses by using our hybrid capture sequencing approach.

Association testing for the five candidate variants with Fisher’s exact test showed three loci that were not linked to the roan phenotype in this larger sample set, and which were therefore discarded (all *p* > 10^−10^ with odds <6). Furthermore, the allele frequencies of the excluded variants exceeded 25% in both roan and non-roan horses. The remaining two variants were present in 75% of the roan horses without an *RN1* or *RN2* haplotype. The SNP chr3:79428717 (T > G), about 100 kb downstream of the *KIT* gene, was present in 5% of non-roan horses, whereas SNP chr3:79656505 (G > A), approximately 38 kb upstream of the *KIT* gene, was only present in *RN3* horses. These two assays showed strong association with the roan phenotype (*p* < 10^−15^ and odds of >20) using Fisher’s exact test, and were assigned roan haplotype *RN3* (Table 2). This roan haplotype, as defined by the presence of at least one copy of each of the two variant alleles, chr3:79428717G and chr3:79656505A, (G-A- vs. TTGG) had *p* < 10^−15^ with odds of >60 using Fisher’s exact test. Using Plink (1.90b7.1), the OR for chr3:79428717 (*p* = 1.1 × 10^−30^) was 15.35 (95% Confidence interval: 8.48–27.79), and, for chr3:79656505, the OR was impossible to measure (*p* = 6.529 × 10^−25^), as it was 100% correlated to the roan phenotype; the r^2^ value was 0.772 for the allele combination of GA vs. TG, based on a small number of samples, and only r^2^ = 0.009 for the assumption that GG vs. TA were linked. Based on these data, both variants are in clear linkage disequilibrium with the *RN3* phenotype, and together can be used as markers for the *RN3* haplotype.

**Figure 1:**
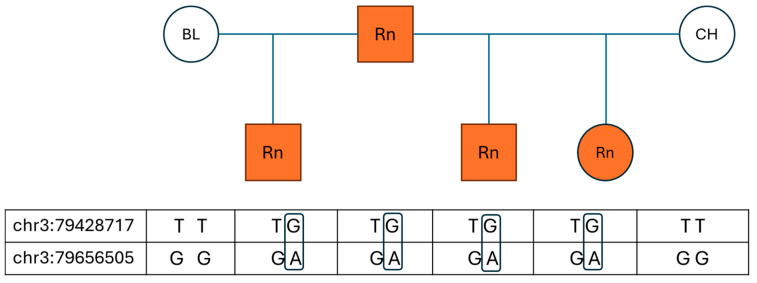
Three horses born to a roan sire and two different non-roan dams show that sire and roan offspring share the two variant alleles, whereas both dams are homozygous wild-type. Offspring can only derive the variant alleles from the father on a single parental chromosome, proving that they are linked. BL = black; CH = chestnut; Rn = roan.

To observe the pattern of inheritance, a small number of available parental animals were added to the study. For five horses, data from both parents was available, and for another six horses, genotype data for one parent was available. For each of these eleven horses, one parent was roan and the other parent was non-roan based on pedigree information and photos. In all 11 cases, the roan parent and the roan offspring shared the variant alleles for both assays, whereas the non-roan parents were all wild-type for the two variants observed (Figure 1). The pedigree of three roan horses revealed a common roan stallion on both the maternal and paternal sides. Interestingly, the non-roan parent in these pedigrees descended from non-roan offspring of this stallion, while the roan parent originated from roan offspring of the same sire. Therefore, the variant alleles seem to be co-inherited from the roan parent and are only present in roan horses, and all non-roan horses possessed only the wild-type genotype (GG) for chr3:79656505 (G > A), while less than 5% of non-roan horses possessed a variant call (TG or GG) for chr3:79428717 (T > G). This genotype distribution is consistent with the hypothesis that the two variants are in phase and that roan is inherited as a dominant trait.

The newly discovered roan haplotype, Roan 3 or *RN3,* was seen in 60 (75%) of the roan samples without a previously described roan haplotype. This *RN3* haplotype was found in American Quarter Horses and American Paint Horses only. Therefore, this haplotype can be considered a relatively young haplotype. This haplotype (*RN3*) is strongly tagged by a G > A substitution at chr3:79656505, a variant not present on the horse reference genome, and which may constitute a private variant in this specific population. We examined the extended pedigrees of several American Quarter Horses carrying the *RN3* haplotype, and, assuming that roan is inherited as a dominant trait, we observed that the roan haplotype *RN3* could be traced to the “Kitch roan mare” (born 1901; AQHA registry U0073276). Current major Quarter Horse sires transmitting this roan *RN3* haplotype are Zippos Mr Good Bar (born 1984) vs. Code Blue (born 2007) and vs. Code Red (born n2007). All 60 horses tested with this haplotype were heterozygous for *RN3*. As we expect this haplotype to be more recent, it is represented by one sire line only, it is not as widespread in the population, and, therefore, we likely did not find any homozygous *RN3*/*RN3* horses as of writing.

Taking the newly discovered *RN3* haplotype together with the previously validated two haplotypes, *RN1* and *RN2*, it is estimated that these three haplotypes explain 85–90% of the roan phenotypes among the samples tested. Of the last group of 80 unknown roan samples tested for this publication, there remains 20 horses with an unknown roan haplotype present. These 20 horses were divided over 10 different breeds (Table 3). Thus, it is expected that there are at least several additional roan variants present but not accounted for.

As with Dominant White, which comprises over 35 haplotypes, of which several haplotypes were identified in only a single individual or a small family as a result of sporadic mutations, we expect that the roan phenotype is also due to multiple independent haplotypes. It is our intention to sequence a number of unknown roans in other breeds to discover their specific roan haplotype.

This exploratory study, using whole genome sequencing of a small number of roan samples, showcases the ease in which new variant alleles associated with the roan coat color can be discovered. Whole genome sequence data allowed us to examine the expansive intergenic regions neighboring *KIT*. For the current report, the limited number of non-roan horses examined using WGS increased the likelihood of variant dropout due to chance missingness of low-frequency variations in the population. By utilizing more broad sampling with WGS in the future, we hope to limit that dropout.

## 4. Conclusions

As proposed in our previous paper [5], we used whole genome sequencing to generate an average of 40× coverage for a sample of horses that included some unknown roan variants. The whole genome sequencing (WGS) of several RN1 and RN2 samples allowed us to re-analyze the *KIT* region, confirming the previously detected roan variants, while performing WGS on unknown roan samples provided new variants, defining a third roan haplotype, *RN3*. Together with the roan *RN1* and *RN2* haplotypes discovered previously, we estimate that these three haplotypes account for the vast majority (~90–95%) of roan phenotypes in American Quarter Horses and American Paint Horses, and all three haplotypes were likely proliferated by popular stallions. As such, we expect the majority of roan horses in the American Quarter Horse population to be assigned one of these three haplotypes. Overall, the *RN3* haplotype is likely present in <1% of all horses. As stated in the previous study, these haplotypes are in linkage disequilibrium with roan, and may not be the causative variant. As the *RN* haplotypes extended beyond the borders of the *KIT* gene, whole genome sequencing is the most logical and cost-effective method to investigate the variants associated with roan.

For other breeds, such as Arabian, Belgian Draft, Clydesdale, Gypsy Cob, Tennessee Walker, and Shire horses, more research into these roan phenotypes is needed, and this is ongoing. So far, only *RN1* is detectable in these breeds, covering approximately 50% of the roan horses in these breeds. Therefore, other roan haplotypes might yet be discovered.

## Figures and Tables

**Table 1:** Samples examined using whole genome sequencing.

Haplotype	Number of Horses
*RN1*/*RN1*	5
*RN2*/*RN2*	1
*RN1*/*RN2*	2
*RN2*/*n*	1
Unknown roan	2
Control (non-roan)	5

**Table 2:** The newly identified *RN3* haplotype’s defining variants.

Roan Haplotype *	Allele 1 (Ref)	Allele 2 (RN)	AF ^†^ Allele 2 (Rn/Non-Rn)	Accession Number
RN3				
chr3:79428717	T	G	0.494/<0.05	rs1142018742
chr3:79656505	G	A	0.494/0.00	No rs ID

* All positions on EquCab3.0. ^†^ Allele frequencies of the two variants in the roan samples and the non-roan samples.

**Table 3:** Remaining unknown unexplained roan phenotypes in diverse horse breeds.

Breed	# Horses
American Quarter Horse	6
Gypsy Cob	3
Arabian	2
Shire	2
Tennessee Walker	2
Appaloosa	1
Belgian Draft	1
Clydesdale	1
Riesian Cross	1
Mangalarga Marchador	1

## Data Availability

Horse sequencing data are not available due to owner confidentiality.
